# Patient and public involvement and engagement with cardiac arrest survivors

**DOI:** 10.29045/14784726.2022.06.7.1.29

**Published:** 2022-06-01

**Authors:** Alison Coppola, Caroline Halliday, Mark Jones, Richard Houghton, Mark Johnson, Nigel Sillis, Michelle Johnson, Debra Sillis, Ruth Endacott

**Affiliations:** South Western Ambulance Service NHS Foundation Trust ORCID iD: https://orcid.org/0000-0003-0135-3783; PPIE Working Group; PPIE Working Group; PPIE Working Group; PPIE Working Group; PPIE Working Group; PPIE Working Group; PPIE Working Group; University of Plymouth; Royal Devon and Exeter NHS Foundation Trust / University of Plymouth Clinical School; Monash University

**Keywords:** mixed methods, out-of-hospital cardiac arrest, patient and public involvement and engagement, research methodology, survivors

## Abstract

**Background:**

Patient and public involvement and engagement (PPIE) with cardiac arrest survivors is an essential component of research to strengthen development, design, delivery and dissemination to ensure research priorities are in the public interest and patient friendly. Cardiac arrest survivors and their relatives were engaged in PPIE to help develop the methods of a research study that aims to reduce individual and care process variation during paramedic-led resuscitation.

**Methods:**

This research methodology paper represents the views of seven PPIE representatives and the authors. PPIE representatives included five cardiac arrest survivors and two relatives. Content for the paper was generated by discussion using audio or video call. Notes were taken by the author which included direct quotations generated by the PPIE process.

**Results:**

The PPIE representatives considered research surrounding the decisions made by paramedics to be important. From their first-hand experiences, survivors and their relatives felt that a future research study should focus on patient survival. The decision-making of paramedics was identified as most important to explore. Quality of life before the cardiac arrest was considered important as this may help to inform best-interest decisions. The neurologic recovery of patients was important; however, rehabilitation may be extensive and therefore unachievable within the study timeframe. Relatives highlighted that while incorporating their views during resuscitation was important, gaining consent for research participation was not appropriate.

**Conclusion:**

PPIE added value and helped to develop a future study to reduce variation in the resuscitation decisions made by paramedics. The group identified what is important to survivors and their relatives and the factors they would like paramedics to consider when making a resuscitation decision. By identifying these factors, the PPIE process has helped to drive the research methods where both quantitative and qualitative designs would be appropriate. Issues in gaining research consent during resuscitation were highlighted.

## Background

Patient and public involvement and engagement (PPIE) in healthcare is described by the National Institute for Health Research as essential, to improve the quality of research and to ensure those affected can focus research on what is important to them. PPIE representatives often have personal experience of the research area and are therefore able to offer a unique perspective, broader than academics or clinicians, to orientate and refine research in the public interest (National Institute for Health Research [NIHR], 2014). Historically, PPIE with patients with experience of cardiac arrest and resuscitation was challenging due to the poor rates of survival (Fiori et al., 2020). It was previously stated that essential evidence from a survivor’s perspective was missing (Haywood, 2016). It is essential that barriers to PPIE are managed, as the process is integral to map and prioritise research in the public interest, and to strengthen the development and design of research and the delivery and dissemination of results (NHS Health Research Authority, 2021). As an exemplar of PPIE, the core outcome set for cardiac arrest in adults (COSCA), a partnership advisory statement between the International Liaison Committee on Resuscitation, patients, clinicians and researchers developed a consensus of outcomes for effectiveness studies on cardiac arrest. The COSCA statement advised that outcomes should include survival, neurologic function and quality of life. This PPIE demonstrates the importance of involving cardiac arrest survivors and their relatives, who offered a unique perspective to increase the quality of a future research study.

### PPIE informing future research

Considering the valuable contribution PPIE makes to research, PPIE with cardiac arrest survivors and their relatives was planned in the early development of a research study that aims to reduce variation in the resuscitation decisions made by paramedics. The study hopes to bridge the gaps in research for which patients should have resuscitation terminated at the scene of the cardiac arrest and which patients should be conveyed to hospital.

This work is important, as the out-of-hospital cardiac arrest outcome (OHCAO) report project identified a potential cause for poor survival in the United Kingdom when compared to other countries. Regional disparities between ambulance services were found, with several reasons identified: individual practice variation, the care process, structure of ambulance services, patient case-mix and the reporting of data (Perkins & Brace-McDonnell, 2015). Ambulance services are comprised of 10 trusts according to NHS strategy. Consistent reporting of data is now established by the introduction of the national OHCAO registry. Individual practice and care process variation remain unexplored, with variables of interest identified as the quality of resuscitation, resuscitation decisions and the response times of ambulance services. To help identify the priorities surrounding this research topic, early engagement from PPIE representatives was sought.

## Methods of PPIE

The PPIE aimed to inform the design of a future study, to identify, prioritise and focus outcomes using the experiences of cardiac arrest survivors. This process hoped to orientate and drive the research methods by identifying important data to be collected and analysed from a survivor’s perspective. This research methodology paper describes the PPIE process and how the involvement of survivors and their relatives contributed to and improved the research design.

### PPIE process

This research methodology paper provides the views and recommendations from the PPIE representatives and authors. Content for the paper was generated by discussion using audio or video call. Individual discussions took place due to the sensitive nature of the topic area in place of a group event. As recommended by PPIE guidance from the NIHR (2016), notes were taken by the author which included direct quotations generated by the PPIE process. PPIE does not require ethical approvals for research design (Couper et al., 2019; INVOLVE, 2018). The PPIE representatives reviewed this paper and provided written consent for the use of quotations and publication.

### PPIE representatives

Discussions with five cardiac arrest survivors, two of their relatives and the lead author took place between October and November 2020. Discussions lasted between 60 and 90 minutes. One survivor was female and four were male. Three were over 60 years and two over 50 years. One survivor had an initial rhythm of pulseless electrical activity (PEA), four survivors had a shockable rhythm of which two transitioned to PEA with a resuscitation duration of 45 minutes. Four survivors underwent significant periods of rehabilitation; however, all felt they had a good or acceptable quality of life following their out-of-hospital cardiac arrest (OHCA).

## PPIE results

The personal views and experiences provided an excellent perspective to develop several themes around research priorities, factors for data collection and outcome measures.

### The research priority: focus on survival

The PPIE representatives felt that research should focus on patient survival. They provided several examples from their personal experience as to why this research focus was important to them. Most survivors met the ambulance staff responsible for their resuscitation. One survivor recalled a conversation as to why the paramedic continued to resuscitate them. They discussed several important cardiac arrest factors which informed this decision: age, fitness, witnessed collapse, immediate bystander resuscitation, while considering the prolonged resuscitation time and a change in rhythm to PEA following defibrillation. The paramedic felt their clinical judgement, underpinned by their own moral values, made them continue resuscitation.

I asked the resuscitation team why they kept going, they said I was young and fighting, they wanted to give me the best chance, it wasn’t a clinical decision, it was a moral decision.

This focus on survivability was not experienced by all. One relative who watched the resuscitation asked the paramedic to continue as they felt this provided the best chance of survival and recovery. They felt ambulance and hospital staff had a negative attitude. This may have been because the survivor presented with factors associated with a poor outcome – non-shockable rhythm, and unwitnessed and long resuscitation duration. The relatives recognised that it was possible staff attempted to manage their expectations as survival was uncertain; however, hope was important to them.

### Quality of life

Survivors spent time in the intensive care unit following hospital admission, with the majority experiencing physical and well-being challenges to their recovery. One survivor suffered a severe hypoxic brain injury, unable to hold a spoon, stand or walk. They felt their quality of life was unacceptable; they were fully dependent and assessed as having no capacity, and did not know that with time they would recover.

Quality of life would be unacceptable if I was not able to do all the things I did before.

Another survivor described feeling physically impaired, their ‘lowest ebb’, but mentally as strong as ever. As their physical health improved, at times their mood lowered. Time and recovery were common concepts with most survivors. Symptoms such as memory loss or fatigue improved with time and therefore so did their quality of life. The survivors had a different level of acceptability regarding their quality of life. No survivor wanted a ‘meaningless existence’. Only one survivor reported the same quality of life prior to their cardiac arrest. The remaining survivors are mostly happy with their quality of life; however, they continue to work towards a full recovery. Quality of life before a cardiac arrest was said to inform the resuscitation decisions made by paramedics.

### The likelihood of recovery

All survivors prior to their cardiac arrest reported good health and an active lifestyle. Four survivors suffered a cardiac arrest during or shortly after exercise. Most survivors reported their cardiac arrest as life-changing and questioned ‘why me’; they could not understand why their cardiac arrest had happened.

He had a few broken ribs, felt really tired and exhausted and kept thinking why did this happen to me, it all felt confusing, he couldn’t understand, he didn’t really drink, smoke, had a normal BP and low cholesterol.

One survivor thinks about their cardiac arrest every day. They wish for just one day not to think about it and to live life as they did before. One survivor described feeling as though they were no longer the same person. They were quieter and worried their loved ones would not like the ‘new me’. Time was needed to aid recovery, and most symptoms, such as memory loss, reduced over time.

### Loved ones

Not all cardiac arrests were witnessed; however, bystanders, family or friends provided basic life support to the survivors. The care delivered during and post resuscitation was often driven by the hopes of loved ones. Loved ones reported their experiences as highly stressful.

I was not in a position to make decisions, it was too emotional, I wanted to get to hospital and if another event happened, then I’d let go.

Relatives wanted the resuscitation to continue. They felt too emotional to make decisions about stopping resuscitation. Little reassurance was given from the medical staff. One relative took great comfort in knowing everything that could be done was; there were no ‘what ifs’ should their loved one not survive. When asked about making decisions during resuscitation, relatives reported feeling too emotional to make an informed decision. In terms of consenting to research participation, the PPIE representatives felt gaining consent was inappropriate and not required; however, consent should be gained after the event if possible.

## Discussion

### Orientating future research

The value of including survivor experiences in the early stages of this study was highlighted by their focus on survivability, orientating the study on what is important to survivors. The survivors highlighted factors that paramedics should consider, and many of these factors were previously investigated to prognosticate survival (Nolan et al., 2006). The group identified that the cardiac arrest factors of age, fitness, witnessed collapse, immediate bystander resuscitation, prolonged resuscitation, conversion of cardiac arrest rhythms and the clinical judgement of paramedics were important data to collect.

Quality of life before and after the resuscitation and how the quality of life informs the decisions made by paramedics was also highlighted as important. Data on frailty and quality of life were limited as this information is not routinely collected. These data will be collected along with routine data as guided by the COSCA statement to make associations between the factors of cardiac arrest identified and the survival of patients. Challenges to collecting quality of life data were previously identified in a review that found measures in OHCA patients were not validated (Haywood et al., 2018). A previous study applied the *health-related quality of life, Charlson’s weighted index of morbidities* (Hellevuo et al., 2018); however, this was only suitable in survivors who were able to reflect before cardiac arrest and resuscitation, not in deceased patients. A scale typically used within UK ambulance services as a measure of quality of life is the Rockwood frailty score which determines patient function, morbidity and cognition (Sternberg et al., 2011). A scoping review on the frailty scale found that while its use within the pre-hospital setting was limited, the scale was predictive of patient outcomes including mortality (Church et al., 2020). Quality of life data will therefore be collected using the Rockwood frailty score. The likelihood of recovery was a key concept from the PPIE representatives; however, prognosticating recovery in the pre-hospital context is challenging. To allow for a better understanding, perceptions of recovery will be explored in the lived experiences of paramedics, in addition to collecting data on the neurologic recovery of survivors.

### PPIE informing research methods

Important data for collection were identified by the group and this has driven the study methods. Quantitative data will be examined in a prospective cohort study. Data collection will include routinely collected data comprising clinical, patient and system factors, frailty score and extent of neurologic disability at 30 days, guided by the COSCA statement. The study outcome measures were agreed as: (1) termination of resuscitation at the scene of the cardiac arrest; (2) patients transported to hospital with ongoing resuscitation but not surviving to 30 days; and (3) patient transported to hospital with ongoing resuscitation with survival to 30 days.

A qualitative enquiry will explore the clinical decision making of paramedics during resuscitation with a focus on family wishes, perceived chance of recovery, health prior to collapse and the clinical judgement and ethical considerations of paramedics. Given the sensitive nature of resuscitation decisions, semi-structured interviews will offer paramedics the flexibility to expand on answers and ask unplanned questions (Clarke & Braun, 2014). Interpretive phenomenology analysis will provide a detailed examination of the paramedic’s lived experience.

As this study will employ two research methods, the parallel convergent mixed methods design will be applied, a suitable design to explore the factors which impact and influence resuscitation decisions for patients with OHCA.

### The value of PPIE

**Figure 1:**
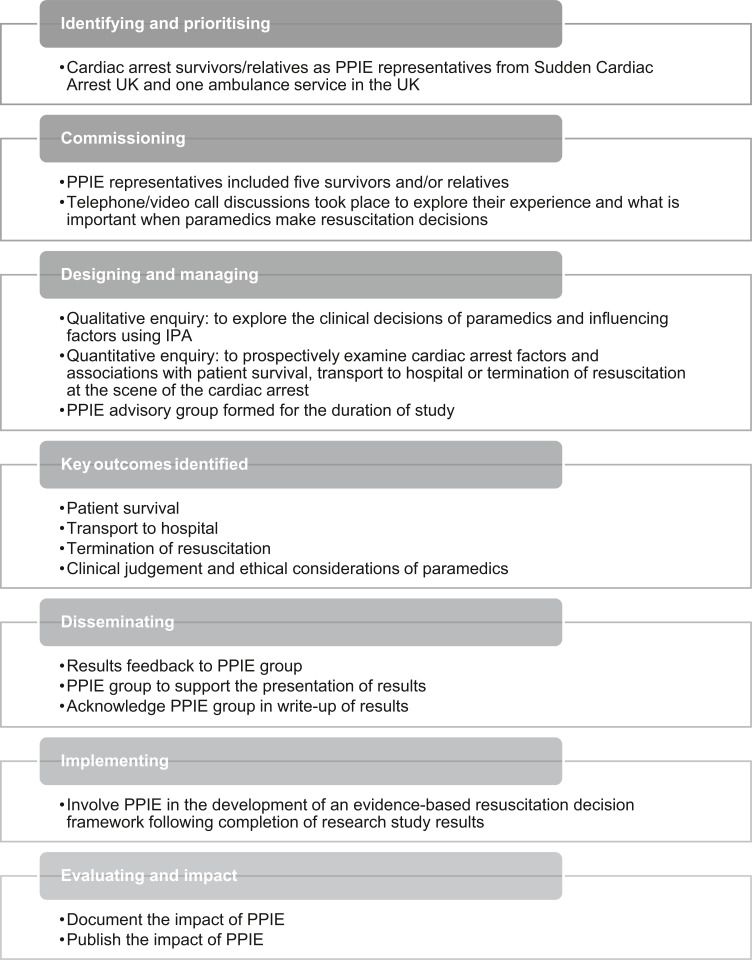
Figure 1. Research process.

PPIE has provided valuable insights from those with experience of OHCA and the findings have been firmly embedded into a study that aims to reduce individual and care process variation in the resuscitation decisions made by paramedics (Figure 1). The National Health Authority suggests that research which involves PPIE is more relevant, designed in an acceptable way for patients and provides a valued research experience (NHS Health Research Authority, 2021). This was certainly our experience of PPIE. Often cardiac arrest research has focused on termination of resuscitation due to the poor rates of survival; however, no criteria have been validated due to finding unexpected survivors (Bossaert et al., 2015). By including the multiple experiences of cardiac arrest survivors and their relatives, the right people, with lived experience, were included in the research process. This in turn makes for more meaningful research, the ultimate goal for involvement as a national standard (Matthews et al., 2019). The PPIE experiences were relevant to ensure we fully understood what matters to patients when paramedics make resuscitation decisions. PPIE is strongly encouraged for this reason, making sure the topic area is of interest and more than a tick-box exercise (Troya et al., 2019).

PPIE was particularly helpful in identifying barriers to consenting patients or gaining consent from relatives during resuscitation. Consent was previously found to be a barrier in emergency care research, with 32.4% of patients believing data should only be accessed with consent (de Tonnerre et al., 2020). The PPIE representatives felt consent during resuscitation was not appropriate. Relatives said they felt too emotional to make informed decisions regarding resuscitation, and therefore gaining consent to participate in a research study at that time would not be an informed decision. They felt it was acceptable to enrol patients into the study without consent; however, if possible, consent should be gained after the event. Deferred consent methods were preferred by the group. This method of deferred consent was successfully employed by the ARREST trial, an OHCA study where informed consent was waived until consent could be sought by the patient or nominated person (Patterson et al., 2018). A similar consenting method would seem appropriate for our study. To mitigate collecting the data in patients unable to consent, an application to the confidentiality advisory group, Health Research Authority, will be submitted.

### Limitations

The neurologic outcome of patients will be collected using the modified Rankin Scale. Outcomes following cardiac arrest are recommended from COSCA at 30 days, 90 days and at regular intervals up until one year (Haywood et al., 2018). Due to the time constraints of this study, not all interval measures are possible and therefore the modified Rankin Scale at 30 days will be reported only. PPIE identified that the neurologic status of the patient may change over time, therefore assessing neurologic outcomes in the short term may not be a true reflection of long-term disability.

## Conclusion

PPIE added value and helped to develop a future study to reduce individual and care process variation in the resuscitation decisions made by paramedics. The group identified what is important to survivors and their relatives and the factors they would like paramedics to consider when making a resuscitation decision. By identifying these factors, the PPIE process has helped to drive the research methods where both quantitative and qualitative elements in a mixed methodology would be required. Issues in gaining research consent during resuscitation were highlighted.

## Acknowledgements

Thank you to Paul Swindell, Sudden Cardiac Arrest UK; Sharifa Hashmen, patient engagement manager and Adam Walmesley, communications manager for South Western Ambulance Service NHS Foundation Trust, for assisting in the recruitment of PPIE representatives. This work was only possible thanks to the survivors and their relatives. Your honest conversation, time, continued support and advice are motivation enough, thank you.

## Author contributions

AC undertook the PPIE activities to inform and orientate future PhD funding. PPIE working group members contributed to the content. AC collated the content and edited the manuscript, supervised, revised and edited by RE. The manuscript was second reviewed by the PPIE working group for validation and edited accordingly by AC for publication. AC acts as the guarantor for this article.

## Conflict of interest

None declared.

## Funding

Ali Coppola, pre-clinical academic fellow, NIHR301003, is funded by Health Education England (HEE) / National Institute for Health Research (NIHR) for this project. The views expressed in this publication are those of the author(s) and not necessarily those of the NIHR, NHS or the UK Department of Health and Social Care, South Western Ambulance Service NHS Foundation Trust or the University of Plymouth.
